# Prognostic factors of recurrence and neck metastasis in oral carcinomas

**DOI:** 10.12669/pjms.326.10661

**Published:** 2016

**Authors:** Behcet Sahin, Suphi Bulgurcu, Ilker Burak Arslan, Ibrahim Cukurova

**Affiliations:** 1 Behcet Sahin, Department of Otorhinolaryngology, Tepecik Education and Research Hospital, Izmir, Turkey; 2 Suphi Bulgurcu, Department of Otorhinolaryngology, Tepecik Education and Research Hospital, Izmir, Turkey; 3 Ilker Burak Arslan, Department of Otorhinolaryngology, Tepecik Education and Research Hospital, Izmir, Turkey; 4 Ibrahim Cukurova, Department of Otorhinolaryngology, Tepecik Education and Research Hospital, Izmir, Turkey

**Keywords:** Tongue cancer, Metastasis, Invasion, Magnetic Resonance imaging

## Abstract

**Objective:**

To assess the effects of tumor size, proximity to midline and invasion depth of oral cancer of the tongue (TC) on neck metastasis and recurrence.

**Methods:**

In this retrospective observational study, was conducted through a chart review of the 11 male and 9 female patients who underwent surgeries with the diagnosis of tongue squamous cell carcinoma and at least one side neck dissection. We wanted to assess effects of tumor size, proximity to midline, and invasion depth of TC, according to the surgical specimens and pre-operative magnetic resonance imaging, on neck metastasis and recurrence between 2007 and 2014. The study was conducted in a training hospital-based otorhinolaryngology clinic. Statistical analyses were performed to determine possible relationship between such tumor features and tumor recurrence and neck metastasis.

**Results:**

Statistically significant relationship were detected between recurrence and the proximity of tumor to midline (p=0.031) and between invasion depth and neck metastasis (p=0.017). No relationship was found between tumor size and recurrence and neck metastasis (p=0.721 and p=0.827, respectively).

**Conclusions:**

Parameters like invasion depth and tumor proximity to midline might provide useful information about prognosis and may help to determine a treatment schedule in patients suffering fdrom cancer of the tongue. The present TNM classification might not be sufficient to provide enough information to determine prognosis and staging adequately in these patients.

## INTRODUCTION

As oral cavity cancers are the second most common tumors following larynx cancer of head-neck region with a rate of 14.1%, cancer of the tongue (TC) constitutes the majority of these tumors, with a rate of 39%. Tongue cancer is frequently seen worldwide due to very common use of smoking and alcohol.^1^ Local and regional recurrences are the main reasons of treatment failure.^2^

The most common known factors affecting the prognosis of TC are, tumor size, tumor proximity to midline, tongue base involvement, cervical lymph node involvement and pathological parameters (invasion depth, status of surgical margin, differentiation, lymphovascular and perineural invasion etc.) detected in surgical specimen or biopsy material.^3^ The major prognostic factor among these parameters is the existence of cervical lymph node metastasis.^3^

Although a myriad of studies have been published on neck metastasis and recurrence of oral cancer of the tongue, available prognostic data, to our knowledge, do not perfectly show potential candidates of recurrence and metastasis from TC.^4^-^6^ Given this situation, the present study was aimed to determine the effects of several factors on recurrence and metastasis of cancer of the tongue in a cohort of these patients from a single otorhinolaryngology center.

## METHODS

In this retrospective observational study, we conducted a chart review of a training hospital-based otorhinolaryngology clinical practice to assess effects of several clinical, radiological, surgical and pathological factors of cancer of the tongue on neck metastasis and recurrence at Tepecik Education and Research Hospital, Izmir, Turkey between 2007 and 2014. The study protocol was approved by the institutional ethics committee of Tepecik Education and Research Hospital. The study was conducted according to the WMA Declaration of Helsinki - Ethical Principles for Medical Research Involving Human Subjects.

The retrospective review included the patients who underwent surgeries with the diagnosis of tongue squamous cell carcinoma (SCC) and at least one side neck dissection. The patients who had a previous surgery, and tongue base tumor or received chemotherapy and/or radiotherapy previously were excluded. For tumor classification, 7th version (2010) of AJCC TNM classification was used.

Tumor size, proximity to midline and amount beyond midline, and invasion depth of cancer of the tongue, and contralateral or ipsilateral recurrence and neck metastasis. Details were recorded according to the surgical specimens and pre-operative magnetic resonance (MR) images of the patients. Tumor proximity to midline of the patients was considered in three groups according to their MR images. The tumors far from midline more than 10 mm were classified as 1st group, 0-10 mm far from midline 2nd group) and ones passing over midline were 3rd group. Pathological specimens were classified in three groups according to invasion depth as 0-3mm, 4-7 mm and over 7mm.

No power analysis was done. Statistical analysis was performed using a computer software package (SPSS for Windows, version 20.0; SPSS Inc., Chicago, IL, USA). Each value was presented as mean ± standard error, if available. Relationship between neck metastasis and tumor invasion, TNM stage, proximity to midline(mm), with amount of passing over midline (mm) was assessed by Chi-square and Fischer tests. The significance limit in all statistical analyses was adopted as a p<0.05 value.

## RESULTS

There were 11 male and 9 female patients (mean age of 63.65±8.05) included in our study. Mean age for men was 61.82±8.82 years and mean age of female ones was 65.89±6.83 years. According to TNM classification one patient (5%) was stage 1, 12 were (60%) stage 2, 3 were (15%) stage 3 and 4 of them (20%) were stage 4a.

Tumor was the right-sided in 14(70%) cases, and the left-sided in 6(30%), however, it was detected to have passed over midline in 2 of the right-sided, and in 3 of the left-sided cases. Partial glossectomy was performed in 17 (85%) patients (9 bilateral supraomohyoid neck dissection, 8unilateralsupraomohyoidneck dissection), hemiglossectomy and bilateral supraomohyoid neck dissection was performed in 2 patients (10%) and subtotal glossectomy with bilateral supraomohyoid neck dissection was performed in 1 (5%) patient.

According to proximity to midline, ipsilateral neck metastasis was detected in 2 of 9 patients in the 1st group. In the 2^nd^ group, none of the 6 patients had any neck metastasis. Ipsilateral neck metastasis was detected in three of five patients in the 3^rd^ group. One of the four recurrent cases was in the 1st group and 3 were in the 3rd group. The relation between proximity of tumor to midline and recurrence was found to be statistically significant (p=0.031) (Table-I). No significant relation between proximity to midline and neck metastasis was detected (p=0.071).

**Table-I:** Relation between proximity of tumor to midline and recurrence.

Proximity of tumor to midline	Patient(n)	Recurrence
>10mm	9	1
0-10 mm	6	0
Passing over midline	5	3

Statistically significant (p=0.031)

When the tumor size (T) of the patients were assessed, T1 was present in 1 (5%), T2 in 12 (60%) of them and T3 in 7 (35%) of them was detected. In 2 of 5 patients with neck metastasis, T3 was detected and three of them were detected to be T2. Two of the four recurrent cases were T2, and two were in stage T3. There were no significant relationship between clinical tumor size and recurrence and neck metastasis (p=0.721 and p=0.827, respectively).

According to invasion depth, there were 5 (25%) patients between 0-3mm, six patients (30%) between 4-7mm, and 9 (45%) patients with invasion depth over 7mm. Invasion depth of five patients with neck metastasis were all detected to be over 7 mm. Invasion depth was between 0-3 mm in one of recurrent patients and it was over 7 mm in other 3 patients. No statistically significant relation between the invasion depth and recurrence (p=0.287). However, a significant relationship between invasion depth and neck metastasis was detected (p=0.017) (Table-II).

**Table-II:** Relationship between invasion depth and neck metastasis.

Invasion depth	Patient(n)	Metastasis
0-3mm	5	0
4-7mm	6	0
>7mm	9	5

Statistically significant (p=0.031)

The follow-up duration of the patients was between six months and six years with an average follow-up of 2.7 years. No recurrence was detected in 16(80%) of the patients during follow up, while recurrence after six months was detected in four (20%) cases (ipsilateral neck recurrence in two, and local recurrence in other two cases).

## DISCUSSION

In this retrospective study, we assessed the effects of tumor size, proximity to midline and invasion depth of oral tongue cancer (TC) on neck metastasis and recurrenc. We found that proximity of tumor to midline is the only related factor with tumor recurrence, while invasion depth is the only related factor with neck metastasis in our cohort. Furthermore, we determined that tumor size was not related with tumor recurrence and neck metastasis. So, we concluded that proximity of tumor to midline was more reliable factor to determine tumor recurrence during postoperative follow-up period, whereas invasion depth of tumor rather than tumor size may be a useful factor to catch the metastatic TC cases, and TNM staging may not always work well in TC patients.

Situation of neck lymph nodes is the most important factor among the prognostic factors in TCs and there is no radiological and/or biological marker that certainly proves their situation.^4^ Hidden neck metastasis rate of these patients was varied in between 15-60%. This wide range is due to the prognostic factors like lateralization, size, invasion depth, perineural or vascular invasion of the tumor and residual tumoral tissue.^5^

Survival rates would be increased by effective treatment applications provided by early diagnosis in TCs that have low survival rates and bad prognosis. There have been many studies about the relation between tumor size and nodal metastasis. According to several studies, lymph node metastasis risk increases if tumor size exceed 2 cm.^6^,^7^ Po Wing Yuen et al. have reported that nodal metastasis risk is increased for the lesions of size larger than 3 cm.^8^ In our study, three of the five patients with metastasis were T2, and 2 were T3, while no significant relation was detected between tumor size and ipsilateral nodal metastasis. Also in comparison of tumor size and recurrence positive patients, two of the four recurrence having patients were T2, and the other two were T3, while there was no significant relationship. But we believe that tumor volume rather than tumor size is a more significant prognostic factor. Thus, tumor volume was measured in a recent study and a significant relation between high volume and the survival plus early recurrence was detected.^9^ Tumor volume measurement couldn’t have been performed due to the lack of device that measures tumor volume in radiology department of our hospital. Because of the lack of contralateral neck metastasis, the relation between tumor size and contralateral neck metastasis couldn’t have been assessed.

As the tumor depth increases, tumor cells can reach to deeper larger vessels.^10^,^11^ This increases the risk of metastasis. In a recent study, it was emphasized that TNM staging is insufficient to determine the prognosis in oral cavity cancers and a new staging system is required including the infiltration depth.^12^ Yuen et al.^13^ have demonstrated that tumor depth is still the most beneficial data about subclinical nodal metastasis, local recurrence and survival, despite all tumor parameters and predictive models. They have showed that the patients having 3 mm tumor depth and had no neck dissection, subclinical nodal metastasis was present in 8%, local recurrence in 0% and 5 years survival in 100% of them. When the tumor depth was between 4-9mm, there was 44% subclinical nodal metastasis, 7% local recurrence and 76% 5 year survival was detected; and they suggested elective neck dissection. Likewise, in another study, high recurrence and metastasis risk was found to be present in tumors with an invasion depth of 4 mm or more.^14^ In our study, it was found to be statistically significant that ipsilateral metastasis risk increases when tumor depth increased. However no significant relation could be found between tumor depth and recurrence. Nevertheless we esteem that tumor depth affects recurrence. As the reason why it was not affected, we considered the aggressive surgery and postoperative radiotherapy applied due to increased lymph node metastasis with tumor depth.

According to various studies, contralateral neck is also a source of nodal recurrence so that region also should be treated in early stage TCs. In a study performed by Lim et al., they have detected pathological metastasis of contralateral neck only in one of 25 (4%) TC patients with clinical N0 stage and detected that none them developed recurrence due to contralateral neck in the follow up period.^15^ In our study, contralateral neck metastasis was detected in none of the 20 patients. Besides, in the follow up, ipsilateral neck recurrence developed in two of four patients developing recurrence and other 2 developed local recurrence in follow up. Also, no metastasis was detected in five patients with tumor exceeded midline.

### Limitations of the study

Firstly, it is retrospective in nature. Secondly, the study has relatively small sample size from a single-center. These shortcomings might reduce the power of the results from our study and limit the generalizability of our results.

In conclusion, our results suggest that proximity of tumor to midline was more reliable factor during postoperative follow-up period to diagnoses tumor recurrence, while invasion depth of tumor rather than tumor size may be an useful factor to catch the metastatic TC cases. Furthermore, we need more data than that of the current TNM classification in order to timely prevent poor outcome in TC patients. In this context, as the further studies grow, new prognostic factors will be detected and clinico-pathological classification and staging would be changed in the future.
